# The Conceptual Development of a Multifunctional Stepladder for Older People and Caregivers

**DOI:** 10.3390/ijerph192114399

**Published:** 2022-11-03

**Authors:** Kah Wei Gan, Poh Kiat Ng, Kia Wai Liew, Yu Jin Ng, Jian Ai Yeow

**Affiliations:** 1 Faculty of Engineering and Technology, Multimedia University, Jalan Ayer Keroh Lama, Bukit Beruang, Melaka 75450, Malaysia; 2 Department of Social Science and Humanities, College of Energy Economics and Social Sciences, Universiti Tenaga Nasional (UNITEN), Putrajaya Campus, Jalan Ikram-Uniten, Kajang 43000, Malaysia; 3 Faculty of Business, Multimedia University, Jalan Ayer Keroh Lama, Bukit Beruang, Melaka 75450, Malaysia

**Keywords:** universal design, older people, caregivers, interior design and furnishings, concept formation, health, quality of life, health services for aged

## Abstract

Stepladders are compact, foldable ladders with flat steps and a platform. Despite all the research and design efforts, there are still limitations in terms of the multifunctionality, usability and simplicity of stepladders and related variants. By combining ideas, features and functions from patent literature, existing products and scientific journals, this study aims to conceptualise a multifunctional stepladder for improved usability. Five concepts are created, which are screened and evaluated against a set of criteria to select the best concept for improved usability, divided into three categories: simplicity, effectiveness and efficiency. The result is a versatile invention that functions as a stepladder, walker, wheelchair and Pilates chair, suitable for older people and caregivers in nursing homes. It allows medical records or supplies to be retrieved from high places without the need for inappropriate aids. The invention can replace wheelchairs and walkers and converts into a Pilates chair to provide a mobile exercise option for older people. The concept offers older people flexibility and independence in terms of mobility and healthcare, while saving space in the nursing home. Further design studies, prototyping and testing are needed before this idea can go into production.

## 1. Introduction

“*So, there’s a movement for simplifying your life: purchase less stuff, own a few things that are very high quality that last a long time, and that are multifunctional*”.

Yvon Chouinard

The importance of multifunctionality can be seen in how much it has streamlined our lives and reduced costs, as stated in the famous quote above. In today’s modern products, multifunctionality is gradually becoming the standard. The concept of multifunctionality refers to the ability to perform more than one function, and has been used in several design studies [1,2,3]. Multifunctional products can reduce clutter by reducing the number of items in a given space. For example, multifunctional furniture can help reduce the amount of storage space normally needed for multiple items, thus improving order in other spaces in use by reducing clutter, which in turn makes the space look more inviting [4].

It is crucial that the design of multifunctional furniture includes space-saving elements or features to optimise energy consumption and thermal comfort while providing aesthetic qualities [5], especially for the ever-shrinking compact homes that lead to more sustainable living [6]. It is believed that the environmental impact of compact homes can be significantly reduced through the use of multipurpose or multifunctional furniture [7] to save more space, as most citizens of densely populated cities in the modern world have to live in compact homes [8]. The multifunctionality of a piece of furniture not only makes it “greener” but also more usable, especially when space is limited.

In the case of nursing homes, it was found that there was overwhelming dissatisfaction among older people with overcrowding in communal areas, as physical separation was not possible in most cases in nursing homes [9]. Living in a confined space with the associated health restrictions aggravated the unpleasant negative sensations and feelings for most of the older people [10]. Privacy is key when residents have the freedom and space to decide for themselves whether or not they want to be alone, have their own routines and create a home-like environment [10]. This can be achieved through a well-designed nursing home environment and the use of multifunctional equipment or furniture. Space for personal items and furnishings facilitates a sense of home or belonging for a more fulfilling everyday life [11]. 

The environmental impact of multifunctional products is also less than the impact of using an equivalent number of single-function products that perform the same number of functions. The creation of multifunctional items is one strategy to lessen the environmental effect of products because less resources are consumed, fewer emissions are produced during production, packaging, transport and distribution and less waste is generated at the end of the product’s life [12]. 

According to the United States Environmental Protection Agency [13], total municipal solid waste (MSW) generation was up to 292.4 million tonnes in 2018. The World Bank estimates that the amount of waste produced in the world will increase to 3.40 billion tonnes by 2050 [14]. Solid waste, household waste and household furniture disposed of in bins and landfills can pollute the environment [15,16,17]. An increase in solid waste generation from household furniture disposal can be due to rapid urbanisation [18], a rise in online shopping [19] and reduced functionality of purchased products [20,21]. Many consumers buy single household items, which leads them to hoard even more products at home. To reduce hoarding, the multifunctionality of household products is essential. An example of a common household product is the stepladder. People often reach for the nearest chair when reaching for something higher, putting themselves at risk of injury and falls [22]. When accessing high levels, a stepladder should be used to avoid even a small fall (under two metres), which can still cause significant injury [23,24]. 

Without the stepladder, there is a risk that users would use other objects to raise themselves, such as chairs or stools, increasing the danger of falling. This finding is common among older individuals in nursing homes [25,26,27]. Common injuries from falling from a chair include broken bones, head injuries, neck and back injuries and internal bleeding [28]. According to Industrial Safety and Hygiene News [29], a total of 500,000 falls from ladders are reported annually in the United States of America and 97% of the falls occurred at home or on farms. These injuries can lead to health problems that negatively impact the nation’s efforts to achieve some of the Sustainable Development Goals (SDGs), particularly SDG 3, which is about the good health and well-being of society.

Unique stepladders and similar variants have been researched and designed [30,31,32,33,34], but there are still many limitations in terms of multifunctionality, usability and simplicity [35,36,37,38,39,40,41,42]. One of the challenges with multifunctional equipment or furniture is the need to be conceptually functional [43]. In addition, the price of most multifunctional products is often very expensive due to their complex design [44,45]. While multifunctionality increases the green added value of a product, attention should be paid to the dialectical measurement of multifunctional furniture, such as whether there is a cost increase that is greater than the increase in functions, or whether there are unnecessary problems caused by the increase in functions [46]. Therefore, it is important for multifunctional designs to be both usable and simple.

There has not been any research on the conceptual design of a multifunctional stepladder for improved usability. Therefore, this study aims to conceptualise such an invention by synthesising ideas, features and functions from the patent literature, existing products and scientific articles. The following research questions (RQs) are proposed to address the problem statement of this study:

RQ1: How can stepladder concepts be proposed based on the synthesis of patent literature, existing products and scientific articles?
RQ2: How can a stepladder concept be multifunctional, usable and simple by design?

## 2. Methodology

The methodology includes the approach to review previous studies using specific keywords related to the aim of the study, followed by a compilation of the strengths and limitations of selected studies. This is followed by a functional screening based on the selected research, followed by concept sketching, screening and scoring of concepts, and final concept generation.

To uncover relevant functions that support the multifunctionality, usability and simplicity of concepts, keywords such as “multifunctional devices”, “multifunctional usable devices”, “multifunctional simple devices” and “multifunctional stepladders” were searched in Google Scholar. The concept of “searchability” was used in the review of sources, which allowed the authors to filter sources consisting of patent literature, technical journal-grade literature, conference proceedings, product literature and textbooks [47]. The relevance of the sources was also determined by the lead author and the second author based on their knowledge and research experience with multifunctional designs. 

The target for the number of sources to be reviewed is around 100 sources. The strengths and limitations of each selected source are highlighted before the functional screening process.

**Figure 1:**
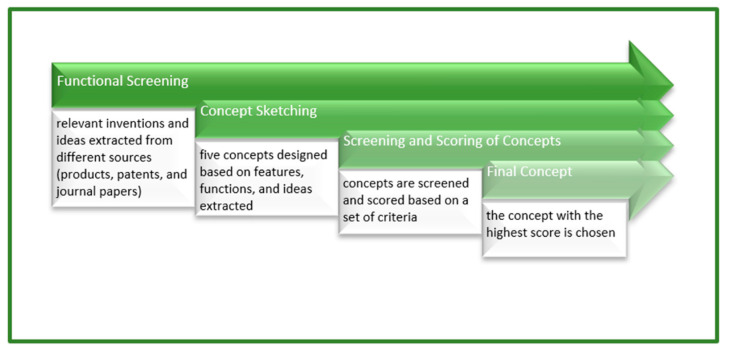
Methodology of conceptual development.

Figure 1 shows the methodology of conceptual development. The methodology includes functional screening, concept sketching, concept screening and final concept selection, which are methods adopted from previous studies [48,49,50,51].

### 2.1. Functional Screening

Functional screening involves extracting relevant inventions and ideas from various sources such as existing products, patents and journal articles. The features, functions and ideas from the functional screening are then combined into a total of five concepts.

### 2.2. Concept Sketching

The five concepts from the functional screening process are sketched using Autodesk Inventor Professional (version 2019). The functionality of each concept is explained in detail.

### 2.3. Screening and Scoring of Concepts

Concept screening helps identify appropriate concepts based on a set of criteria that are used to evaluate the concepts [52,53]. The criteria include multifunctionality, ease of disassembly, foldability, ease of storage, time saving, efficiency, simplicity, effectiveness and durability. The reasons and motivations for the chosen criteria are as follows:

Multifunctionality: As an example, the criterion for concepts that fulfil more than two, three, four or five functions is selected as one of the criteria to be evaluated. The principle of multifunctional design encourages designers to look for multiple functions and opportunities to combine as many functions as possible and ensure that the design fulfils all functions as effectively as possible [54]. 

Ease of disassembly and foldability: The ability to easily disassemble a design makes it easier to repair or upgrade the invention, extending its useful life [55]. It can also help with recyclability, as the degree to which an invention can be easily disassembled determines what the end of life will look like. Apart from this, foldability is also an important aspect for bulky inventions as it improves the portability of the invention. 

Ease of storage: The ease of storing an invention is also important in solving the problem of lack of space in homes. Space problems faced by homeowners, such as lack of work space, garage space, kitchen space and even play space, affect the quality of life of householders [56]. Ease of storage is an important factor in the development as it takes into account the downsizing of the invention to reduce the space required in the home.

Time saving: Saving time is also important for users. An example of an invention that saves time is the hair dryer. Users would buy a hair dryer to dry their hair because it saves a lot of time compared to drying hair manually with a towel. A survey found that 36% of users who rely heavily on time-saving products and services often do so to reduce stress, simplify everyday tasks and save time for other activities [57]. 

Efficiency, effectiveness and simplicity: Usability is sometimes also referred to as user-friendliness. Usability can be achieved by understanding user requirements, formulating usability goals and selecting the best techniques for usability evaluations [58]. This study considers the formative conception of usability, where the presence of usability depends on the absence of usability problems [59]. In this study, simplicity, effectiveness and efficiency were selected as criteria for concept screening because these factors serve as goals for improving formative usability. Simplicity in design refers to the act of minimising, refining or editing a design [60]. According to ISO 9241-11:2018 [61], effectiveness and efficiency can be defined as follows:

- Effectiveness: Completeness and accuracy with which users achieve specific goals.
- Efficiency: Resources used in relation to results achieved (common resources include human input, time, materials and costs).

Durability: The durability of an invention is important because it is the economic aspect of the invention. Cheaper and less durable products are prone to frequent damage, which means that repair or replacement can be more expensive [62]. A durable product is more environmentally friendly because when a product is durable, it lasts longer, so it has more time to perform its function and, to some extent, the environment has more time to regenerate the materials used to make the product.

In the concept screening stage, one of the concepts is selected as a reference. The other concepts are given the symbol “+” if they are able to outperform the reference against the criteria. The symbol “−” is given to the other concepts if their ability is below the performance of the reference based on the criteria, and the symbol “0” is given to the other concepts if their ability is equal to the performance of the reference based on the criteria. Based on the negative and positive scores, the net score is calculated and the three concepts with the highest net scores are selected for further evaluation.

Similar criteria (multifunctionality, ease of use, simplicity, time saving and ease of storage) were chosen for the evaluation of the remaining concepts, as the above criteria are important for the future production of the proposed invention in this study. Safety is also an important criterion to ensure that there are no harmful effects to the user. Safety is the ability of a product to be safe for its intended use, which is determined when it is evaluated against a set of established rules [63]. Convenience of a product is another criterion to consider, as it includes the ease with which the product can be carried and transported, supported by its foldability, ease of assembly and space-saving features.

### 2.4. Final Concept

The lead author of this study conducted the screening and scoring with some assistance from the co-authors. However, the lead author alone recommended the ratings for each concept based on his particular expertise and familiarity with various multifunctional devices. The lead author was also at the forefront of the design of this study and had a solid understanding of the cost and prototype factors that needed to be considered. The co-authors of this study agreed with the lead author’s assessments, as he has an excellent grasp of multifunctional designs. Similar procedures were also used in other studies [64,65,66]. For further evaluation of the concepts, the following formulation is used for the weighted score (*WS*) that a given concept achieves for each criterion. This formulation has been used in other similar studies before [64,67]. A four-point scale is used.

(1) $$WS_{j}=\overset{n}{\underset{i=1}{\sum }}R_{ij}W_{i}$$

where

*R_ij_* = rating of concept *j* for *i*th criterion;
*W_i_* = weightage for *i*th criterion;
*n* = number of criteria;
*WS_j_* = total weighted score for concept *j*.

The rating is assigned for each concept according to the criteria. The ratings can be defined as follows:

1. Extremely poor fulfilment of the criterion;
2. satisfactory fulfilment of the criterion;
3. good fulfilment of the criterion;
4. extremely good fulfilment of the criterion.

The concept with the highest score comes first among the other concepts and is selected as the final design that solves the problems in this study.

## 3. Results

This section tabulates and describes the results of the relevant sources screened, followed by a consideration of the strengths and limitations of each relevant source. The results of the functional screening, concept sketching, screening and scoring of concepts and the final concept are also described in detail in this section.

### 3.1. Key Findings from Past Studies Relevant to the Present Study

Using the concept of “searchability”, about 20 relevant sources were selected out of 100 sources for the conceptual synthesis of the functions. The strengths and limitations highlighted in each source are discussed in the next section.

In terms of multifunctional assistive devices, Rui and Gao [41] designed a new multifunctional wheelchair that meets the requirements of safety and stability. The adjustable function of the wheelchair, which enables the transition from wheelchair to bed, facilitates patient transfer and meets the needs of older people, people with disability and rehabilitated patients. The concept of multifunctionality of wheelchairs is suitable for patients who have difficulty walking due to illness, disability, age-related frailty or injury [36]. For example, an additional function of the wheelchair that facilitates the user’s mobility and flexibility when defecating can be very useful for a user who has difficulty walking. 

Apart from wheelchairs, walking aids that are common aids for walking should be developed with multifunctionality aspects in mind, e.g., with a seat function to improve usability and comfort for older people [40]. Multifunctionality in walking aids is important to improve the quality of life of older people because most walking aids support older people in walking but do not consider other factors such as sitting after prolonged walking or standing [39].

Gu et al. [68] designed a walking aid that offers multiple features and functions such as folding and medication storage in a cylindrical structure. The product is designed to provide safety especially for older people and reduce their mobility efforts through the design of a vertical wheel. In developing rollators (a type of walking aid), Costamagna, Thies, Kenney, Howard, Lindemann, Klenk and Baker [35] suggested that the use of a rollator should involve more complex tasks that correspond to daily walking activities. Rollators can serve as assessment tools in clinics for person-specific instruction and training, as well as evidence-based training for fall prevention.

In the search for multifunctional devices, the authors found that there is somehow a connection between multifunctionality and medical assistive devices as described in the previous sections. So, there could also be a link to older people using these devices in care homes. Therefore, in addition to “multifunctional devices”, the authors decided to search for keywords such as “elderly” or “older people”. The results uncovered the need for exercise among older people who were placed in care homes with minimal activity.

In the context of older people, regular exercise is also very important to reduce the risk of hypertension and diabetes. Levinger, Panisset, Parker, Batchelor, Tye and Hill [37] suggested that the participation of older people in outdoor sports activities should be increased by providing them with on-site supervision and guidance in the use of outdoor fitness equipment. 

Researchers who studied Pilates as a method of body conditioning found that this method of exercise is becoming increasingly popular, especially among older patients [69]. This method, which uses springs to increase the load of the exercises to reverse muscle atrophy, was founded by Joseph Pilates when he worked with wounded war patients who were on bed rest [70]. Roller et al. [71] studied the effects of Pilates exercises with a reformer and found that Pilates reformer exercises produced significant improvements in static and dynamic balance, functional mobility and active range of motion in the hips and ankles in adults over 65 years of age. Suna and Işildak [42] found that the physical and physiological characteristics of sedentary women changed positively after regularly performing Pilates reformer exercises. 

In terms of general exercise besides Pilates, adopting yoga postures on a chair at the office can improve several physiological and psychological markers of stress, leading to significant health benefits such as a lower risk of cardiometabolic disease [38]. In the context of multifunctional exercise inventions, Kiser [72] invented a multifunctional exercise bench equipped with a storage space for exercise equipment such as weights, skipping ropes, water bottles, yoga mats, decorative hand weights and resistance bands. Yu [73] also invented a multifunctional exercise machine that allows the user to exercise the hands, feet, lower back and back simultaneously.

Wyatt and Wyatt [74] invented a multifunctional exercise device in which a user couples one (or more) of the plurality of exercise media to the engagement channels, performs at least a first exercise by pulling the coupled exercise media from a contracted position to a stretched position and changes the orientation of the exercise media to perform at least a second exercise. Lin [75] also invented a multifunctional fitness device having an adjustable table that allows different exercise attachments (e.g., pedal unit, left leg unit, sit-up support) to be interchanged according to different exercise requirements.

The keyword search for “multifunctional devices” also found household inventions such as multifunctional furniture. With regard to multifunctional furniture, Wartes [33] invented multifunctional plug-in furniture that can function as an alternative chair, step stool, laundry basket, two-level table, bench, chest, rocking bench, toy box and cradle. Yen and Yang [34] invented a multifunctional folding table that can be placed in a small space in the kitchen, dressing room, laundry room and garage. Lee [30] invented a multifunctional chair that has an extended range of use and can be converted into a deck chair or ladder. 

The keyword search for “multifunctional stepladder” also found tools and hardware inventions with varying degrees of use. On the subject of hardware and tools, Tornabene, Babkes and Howell [32] invented a device that combines the functions of a hand truck, stepladder and dolly to create a versatile device for moving, lifting and supporting. Smith [31] invented a ladder top storage rack, which refers to the attachment to the top of a ladder to keep tools and fasteners within reach. Table 1 summarises the main findings from previous studies that are relevant to the present study.

### 3.2. Strengths and Limitations of the Main Findings from Previous Studies

Rui and Gao [41] designed an invention that converts from a wheelchair to a bed to relieve caregivers in patient care and to meet the needs of the older people and people with disability. Iksan and Susilawati [36] designed a multifunctional wheelchair based on the fuzzy analytical hierarchy process that allows users with disability more flexibility in toileting. An important factor to consider is that the main users of this technology are the caregivers, because it was developed to make their work easier. However, such wheelchairs may not be as flexible or suitable for outdoor activities due to their bulkiness and weight.

Costamagna, Thies, Kenney, Howard, Lindemann, Klenk and Baker [35] pointed out that the use of rollators as assessment tools in clinics could enable person-specific counselling and training as well as evidence-based training. Rollators with multiple functions, such as an additional sitting function, not only help to address usability issues, but also provide greater comfort, ease of use and quality of life for older people [39,40]. Nevertheless, the rollator may not be suitable for those who have problems with balance or weakness when standing. Rollators also require more storage space than other mobility aids with similar functions, such as canes.

Gu, Wang, Sun and Wu [68] designed a walking aid for older people that can perform several practical functions such as folding and storing medicines in a cylindrical structure at the same time. Even so, such a design may not be suitable for use on wet surfaces as there appear to be no locking or braking mechanisms.

Levinger, Panisset, Parker, Batchelor, Tye and Hill [37] suggest that outdoor exercise for older people provides them with more opportunities for physical activity and social contact in a public setting. Nonetheless, outdoor exercise equipment may have limitations in terms of freedom of movement, minimum age, weight and shape or size, which could affect or compromise the safety of older people.

Shedden and Kravitz [69] suggested that the use of springs in Pilates exercises helps to reverse muscle atrophy. Suna and Işildak [42] also studied Pilates exercises in sedentary women and found that the exercises can reduce the risk of hypertension and diabetes, which are common in older people. Then again, the exercises can lead to injury if users do not adopt the correct postures. Improper posture could also reduce the effectiveness of the cardiovascular exercises.

Roller, Kachingwe, Beling, Ickes, Cabot and Shrier [71] mentioned that Pilates exercises significantly improve static and dynamic balance, functional mobility and active range of motion of the hip and ankle. However, the results were not reviewed longitudinally (e.g., 6 months or one year after the intervention) to observe the sustainability of the improvements.

While 15 min of yoga poses on a chair in the office can improve physiological and psychological stress indicators [38], some workplaces may not be as flexible about allowing such a workout in the office. There is also a risk of adopting incorrect postures if one is unsupervised while exercising in the office, which can lead to injury.

Although several multifunctional exercise machines have been developed to facilitate the training of different parts of the body, convenient storage and different types of exercises [72,73,74,75], these inventions can be expensive and bulky to carry around.

Several multifunctional pieces of furniture have been developed that combine the functions of a recliner, stepladder, step stool, laundry basket, two-tier table, bench, chest, rocking bench, toy box, cradle and folding table [30,33,34]. However, these inventions may not be very portable and flexible. In addition, the strength of the auxiliary table could be compromised by the material and the many hinges, so it may be less resilient than a standard table. 

Finally, Tornabene, Babkes and Howell [32] designed a multifunctional invention that combined the functions of a hand truck, a dolly and a stepladder to allow versatile moving, lifting and supporting. Nevertheless, the design seems more suitable for the industrial sector than for domestic or other use. While there is an invention of a ladder that keeps tools and fasteners within reach with a storage rack on top of the ladder [31], it might be difficult to lift or carry such an invention with a storage rack mounted on top. Table 2 summarises the strengths and limitations of the main findings from the previous studies mentioned above.

### 3.3. Functional Screening Results

From the existing products, the idea of the stepladder was adopted for this study because it is a self-supporting device that does not need to be leaned against any kind of support to be used [76]. The idea of the hand truck was also taken up as it is very useful for transporting heavy loads efficiently and without injury [77]. Walking and standing aids are also singled out because these devices are important to support people with walking or mobility impairments [78,79]. The walking and standing aids can also reduce the risk of falls, especially in older people. The function of the chair is singled out because of its importance and frequency in the design of multifunctional furniture [80].

As far as patents are concerned, the ideas of a dolly and a sack truck are used for this study. The dolly and sack truck are combined into a multifunctional concept that can perform tasks such as transporting heavy loads with the sack truck function and transporting large loads with the dolly function [32]. The idea of the multifunctional folding chair that can be transformed into a bed is also extracted [81].

Researchers highlight the idea of a convertible wheelchair-to-bed system because it reduces patient care labour and accommodates the transportation needs of older people and people with disability [41]. In addition, the idea of the medication container in the multifunctional combined walking aid is highlighted because it facilitates the storage of medication [68]. The idea of a foldable wheelchair stretcher is also raised because it allows the conversion of a stretcher into a foldable wheelchair to facilitate manual patient care and transport [66].

There was also the idea of incorporating the function of the Pilates chair into the design process, based on findings from previous studies [42,71]. The Pilates chair is basically a box with one side that can be pushed down against the resistance of springs, like a big pedal [82]. Portable chairs are being developed to make the transition from sitting to standing safer and less difficult for older people, patients with injuries or people with disability [83]. Table 3 summarises the ideas taken from different sources for this study.

### 3.4. Concept Sketching Results (RQ1: Stepladder Concepts from Synthesis of Patents, Products and Articles)

**Figure 2:**
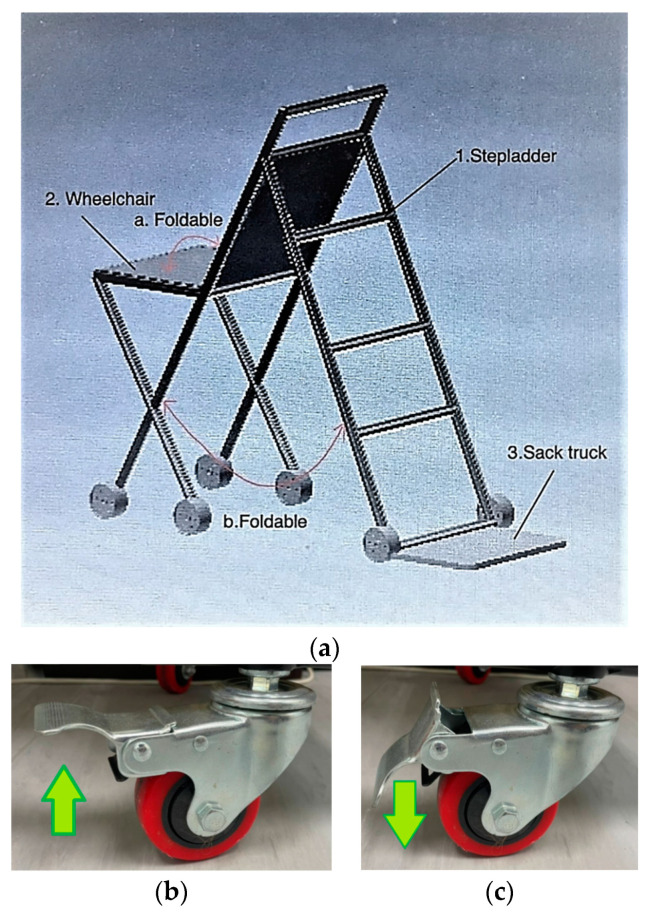
Concept 1. (**a**) Overall view of concept 1. (**b**) Total locking brake freed for wheels to move. (**c**) Total locking brake locked for wheels to be rigid.

Concept 1: For concept 1, the function of the wheelchair is extracted and used in point (2) of Figure 2. The wheelchair is foldable in the direction shown in point (a) to facilitate storage. The mechanism used for the folding function is a hinge mechanism. The wheels of the wheelchair are lockable to increase safety and stability. The wheelchair can be used at home to help injured people or people with disability. The stepladder function is also extracted and used in item (1). The sack truck function is extracted and used in point (3). The stepladder and sack truck are foldable as shown in item (b), using a hinge for the folding mechanism. There is a locking mechanism to lock these features together with the wheelchair so that the mobility of the invention is not hindered even when fully unfolded.

**Figure 3:**
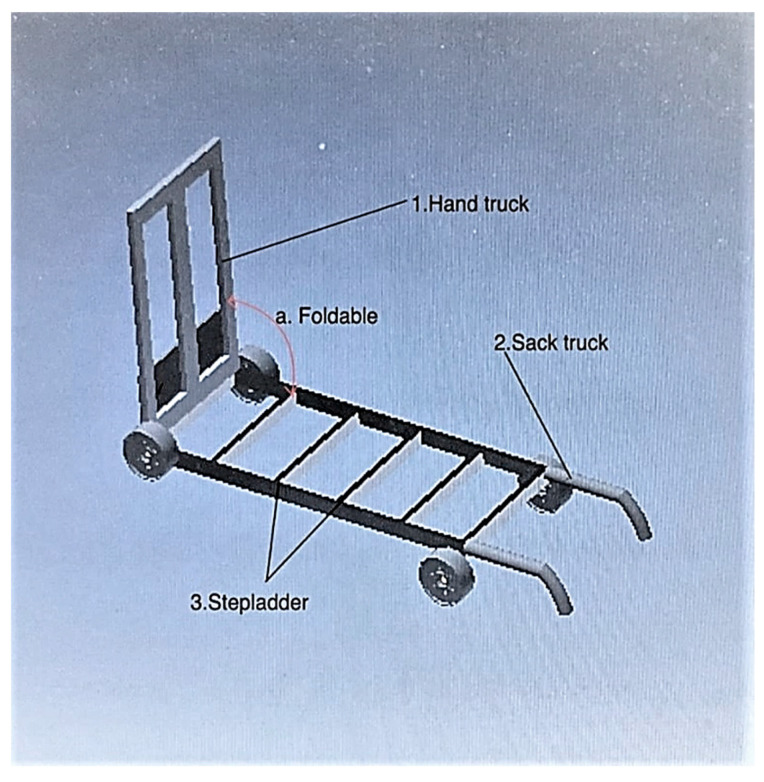
Concept 2.

Concept 2: For concept 2, the function of the hand truck is extracted and used in item (1) of Figure 3. The handle of the hand truck is foldable with a hinge mechanism as shown in point (a). The wheels of the hand truck are lockable so that the user can transport and hold the load without worrying about the stability of the hand truck. The function of the hand truck is extracted and used in point (2). The sack truck can be used by folding the handle of the hand truck towards the wheels in point (a). The stepladder is taken out and used together with the sack truck in point (3). This type of stepladder is usually leaned against a wall so that the user can climb up or down to a higher position.

**Figure 4:**
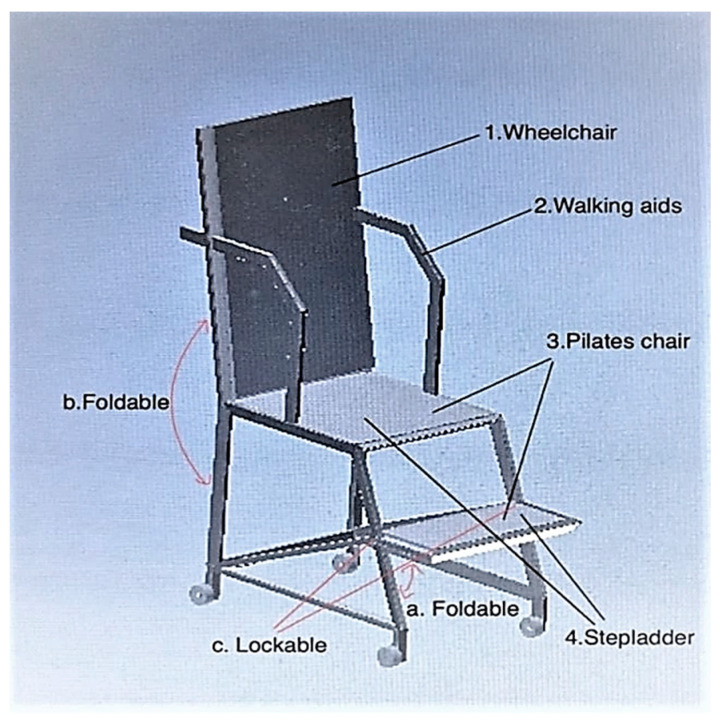
Concept 3.

Concept 3: For concept 3, the function of the walking aid is extracted and used in point (2), as shown in Figure 4. The walking aid is intended to help the user increase lower limb stability during outdoor activities. The function of the wheelchair is extracted and used in item (1). The wheelchair is used by injured users or users with disability. The wheels are lockable to ensure the safety of the user. The function of the Pilates chair is extracted and used in point (3). The Pilates chair helps the user to do exercises that involve postures such as lying, sitting and standing on the chair. The function of the stepladder is extracted in point (4). The step of the stepladder has a locking mechanism so that it is rigid and the user can put all his/her weight on it. The stepladder is normally used as a two-step stepladder, which is primarily intended for users to fetch (or put down) something from a higher place.

**Figure 5:**
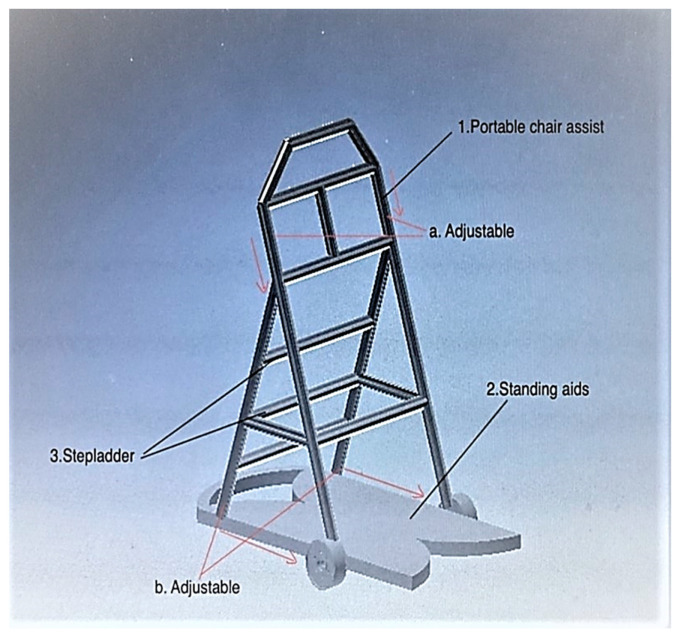
Concept 4.

Concept 4: For concept 4, the function of the standing aid is extracted and used in point (2), as shown in Figure 5. It is equipped with an adjustable footboard that works with a sliding mechanism, as shown in point (b). The user can adjust the board in a way that is comfortable and safe for him/her. The functions of the stepladder and the portable chair aid are extracted and used in items (3) and (1), respectively. The portable chair aid is adjustable to the user’s height as shown in point (a). The stepladder can be used for household tasks such as changing light bulbs, cleaning high places or repairing high places.

**Figure 6:**
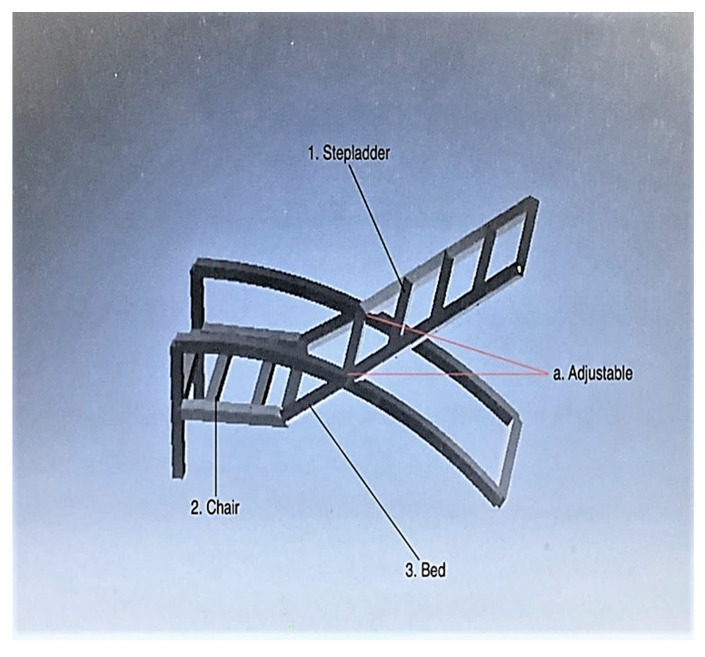
Concept 5.

Concept 5: For concept 5, the chair function is extracted and used in item (2), as shown in Figure 6. The chair is in the form of a lazy chair that can be adjusted to the user’s comfort, as shown in point (a). The functions of the stepladder and the bed are extracted and used in points (1) and (3), respectively. This idea is inspired by the events of the COVID-19 pandemic, where most quarantine stations did not have enough beds and chairs for patients at critical times. The orientations of the bed and stepladder are adjustable to fit the dimensions and requirements of the users.

### 3.5. Results of the Screening and Scoring of the Concepts

Table 4 shows the screening of the concepts against the established criteria. It is important to note that there was no other multifunctional design in the literature or on the market that could serve as a reference for the concepts. The only designs available were designs with one or two functions, which could not serve as a reference due to the lack of multifunctionality. Therefore, concept 1 is selected as a reference because its design seems to be very similar to other designs available in the market, unlike the other concepts. After the screening process, concepts 2, 3 and 4 are selected for further evaluation and comparison.

### 3.6. Final Concept Result (RQ2: Multifunctional, Usable and Simple Stepladder Concept)

The remaining concepts are further evaluated, with more critical criteria such as multifunctionality, usability and safety being weighted more heavily, to see if the previous ranking of the top three concepts would change if these criteria were given more importance. From the evaluation of the remaining concepts in Table 5, the ranking (from best to worst) is concept 3, 4 and 2. Concept 3 is selected as the final concept, with the functions of a Pilates chair, a wheelchair, a stepladder and a walking aid. In principle, concept 3 is a multifunctional stepladder.

## 4. Discussion

In the search for sources, a total of 20 relevant sources of patents and journal articles were found. Based on the consideration of strengths and weaknesses, several ideas were extracted as potential functions for the new invention. These included the stepladder, the hand truck, the walker, standing aid, the chair, sack truck and dolly, the folding chair, the wheelchair stretcher, the medical storage tank of a multifunctional combined walker, the wheelchair bed and the Pilates chair. The synthesis of these functions led to the creation of five concepts. After the screening and scoring process, concept 3 was selected as the final concept.

This concept can be used by older people as well as by caregivers in nursing homes. Even though the older person is supposed to use most of the functions (wheelchair, walker, Pilates chair) and the caregiver seems to use only one function (stepladder), it is important to realise that the older person will still need help from the caregiver when switching between the functions of the invention. Since the invention was developed to make the caregiver’s job easier, this means that the main users of the invention will be the caregivers. This invention is also intended for formal caregivers who work in nursing homes, as it can be difficult to determine the circumstances of different homes and the different training of informal caregivers.

The stepladder helps caregivers retrieve medical records, medications or other items from high places without having to use unsuitable equipment (such as stools or chairs). The rigidity of the stepladder is controlled by lockable wheels at the base of the stepladder and lockable joints at the base of the second step. 

The invention can be converted into a Pilates chair to provide an alternative for older people to exercise and maintain an active lifestyle wherever they may be. The function of the Pilates chair is the key uniqueness and inventive step in this invention, as there are no variants of other wheelchairs, walkers or chairs integrated with the Pilates chair yet. Pilates is an exercise that focuses on strengthening the body, with an emphasis on core strength [84]. The exercise helps to improve the user’s overall fitness and general well-being. This form of exercise was developed by Joseph Pilates in Germany, where he was a carpenter and gymnast. The Pilates exercise can be modified to provide either a gentle strength training or a challenging workout, making it suitable for beginners and advanced exercisers.

The Pilates chair is essentially a box with a padded seat and a pedal attached to one side of the box with springs [82]. The Pilates chair allows for more exercise positions compared to other equipment and provides an effective environment for practising movements while standing, making this chair a highly functional piece of equipment [85].

The second step is a flexible step controlled by springs and used for the function of the Pilates chair. When the function of the Pilates chair is needed, the springs create some resistance when the user moves the step up and down with his or her feet for exercise purposes. The lockable joints resist the flexibility of the second step when the step ladder function is needed. 

The stepladder function is interchangeable with the functions of a walker and a wheelchair, making the caregiver’s work more efficient and comfortable. The walker function is similar to the function of a wheeled walker. The multifunctional attribute, which combines four functions in one device, not only saves costs but also space. Although several environments were involved in the literature synthesis of this study (outdoor, office, farm, domestic, etc.), these different environments were used mainly as a creative approach to consider other contexts for improved patentability. The reason for such an approach was so that the invention would not only be novel, but also inventive. Inventiveness ensures that the novel feature of an invention is not trivial and adds a useful technical effect. The technology readiness level (TRL) is TRL 2 (technology concept formulated) and a patent has been filed (patent application number: PI2022005469, filing date: 3 October 2022) for possible collaborations.

The current design addresses a number of issues brought up by earlier versions. For instance, in contrast to some designs that were intended for industrial usage, its multifunctionality aspect is suited for household application [32]. In comparison to earlier design iterations, the current multipurpose design is also more flexible, portable and less bulky [30,31,33,34]. 

The current technology also ensures that seniors can keep a healthy seated posture while performing basic Pilates exercises with little assistance from carers. By practising Pilates or chair-based yoga activities under supervision, the risk of bad posture and injuries is decreased [38,42]. With fewer limitations on its size, structure and function, the proposed invention also gives senior people the freedom to exercise both indoors and outside, giving them more flexibility in their physical and social activities [37].

However, further testing is needed for the suggested design’s safety component. As an illustration, compared to other multifunctional designs, it might still be risky to operate on wet surfaces or without stopping mechanisms [68]. Although the suggested innovation has a locking mechanism at the wheels, if the older person wants to get up to regain his or her balance, assistance from the caregiver is still needed to manually lock the wheels. As a result, similar to earlier rollator variations [35,39,40], this invention in its rollator-transformed state may still not be suitable for older persons who have balance problems or frailty when standing.

It would be interesting to investigate how this invention can assist caregivers in reducing their stress or mental health state given the evidence of how caring for older people in nursing homes can impact caregivers’ mental health [86,87,88] and the evidence of how assistive technologies lessen the burden among caregivers of older people [89]. Given the impact of robotics on assistive technologies for care homes [90,91], it would also be interesting to explore how robotics can be incorporated into this idea to improve and personalise care for older people. 

### Limitations of Study

One of the limitations of this study is that there are no experimental reviews to capture the different needs and expectations of users in elderly care. An improvement for this study would perhaps be to conduct a survey, focus group or actual site visit and assessment to identify such needs and expectations so that triangulation of data could be carried out to verify the literature synthesis. 

This study’s absence of satisfaction as a usability standard is also one of its limitations. Usability was only taken into account in the current study through efficiency and effectiveness. The next study will, however, include an evaluation of idea satisfaction alongside the testing of the actual prototype. 

The design of the idea itself still needs to be improved. For instance, when using the stepladder function, it is necessary to take into account the incorporation of a grasp support to improve sustained balance. Physical testing of the sitting mechanism when the innovation is used as a wheelchair is also necessary. Furthermore, the invention is still a technological concept and has yet to go through the proper design stages. Further material selection, mechanical design analysis and stress analysis are required before the invention can be prototyped and tested. Tests with older people and caregivers from nursing homes must also be considered. Finally, cost and financial analyses must be carried out to determine the profit and selling price of this invention.

## 5. Conclusions

The aim of this study was to conceptualise a multifunctional stepladder for improved usability by synthesising ideas, features and functions from the patent literature, existing products and scientific articles. Two research questions were proposed to address the aim of the study, namely “RQ1: How can stepladder concepts be proposed based on the synthesis of patent literature, existing products and scientific articles?” and “RQ2: How can a stepladder concept be multifunctional, usable and simple by design?”.

RQ1 was addressed by synthesising ideas, features and functions from patent literature, existing products and scientific articles. The underlying criterion of the conceptualisation was to improve usability, which was divided into simplicity, effectiveness and efficiency. As a result, a multifunctional stepladder was conceptualised with four functions, namely a stepladder, a walking aid, a wheelchair and a Pilates chair. The conceptualisation and evaluation process took into account multifunctionality, usability and simplicity, thus addressing RQ2.

The multifunctional stepladder can be used by geriatric nurses in nursing homes. It enables patients’ files or medicines to be taken from high places, thus preventing the use of unsuitable aids such as stools or chairs. The function of the stepladder is interchangeable with the functions of walkers and wheelchairs, making the work of nursing staff efficient and convenient. The invention can be converted into a Pilates chair to provide older people with an exercise alternative wherever they may be. The idea is not only convenient for caregivers in nursing homes, but also gives older people flexibility and freedom in terms of mobility and healthcare, while saving space in the nursing home.

## Figures and Tables

**Table 1:** Summary of the most important findings from previous studies that are relevant to the present study.

Authors	Title	Sources	Summary
Yen and Yang [34]	Multifunctional Folding Table	Patent	A folding table that can be placed in a confined space in the kitchen, dressing room, laundry room or garage.
Rui and Gao [41]	Design and Analysis of a Multifunctional Wheelchair	Journal Paper	- Designed to meet strength and safety requirements. - The adjustable posture from wheelchair to bed facilitates the work of caregivers and meets the needs of older people and people with disability.
Iksan and Susilawati [36]	Design of a Multi-Functional Wheelchair Based Fuzzy Analytical Hierarchy Process	Journal Paper	- Helps people who have difficulty walking due to illness, limited mobility or injury. - An additional function that facilitates the mobility and flexibility of users with disability in convenient bowel evacuation.
Mohite, Toraskar, Chaturvedi and Bose [40]	Design and Analysis of Advanced Walker Cum Rollator	Journal Paper	- The design of walking aids rarely takes into account aspects other than helping the user to walk. - A walking aid with multiple functions helps to reduce the problems of older people while adding value to them and improving their quality of life.
Mohite, Mollah, Bagchi and Singh [39]	Design of Hybrid Rollator Cum Walker for Elderly: Review on Literature	Journal paper	- Multifunctionality in walking aids is important to improve the quality of life of older people. - Walking aids could have other functions besides assisting with walking, such as sitting after prolonged walking or standing.
Gu, Wang, Sun and Wu [68]	Design of Multifunctional Combined Walking Aid	Journal Paper	- The design of the walker can perform multiple functions, such as folding and storing medicines in a cylindrical structure at the same time. - The walker is designed for older people to ensure their safety and reduce their mobility efforts by using a vertical wheel.
Costamagna, Thies, Kenney, Howard, Lindemann, Klenk and Baker [35]	Objective Measures of Rollator User Stability and Device Loading During Different Walking Scenarios	Journal Paper	- The practice of using the rollator should include more complex tasks representative of daily walking activities. - Rollators can be used in clinics as assessment tools for person-specific instruction and training, as well as evidence-based training for fall prevention.
Levinger, Panisset, Parker, Batchelor, Tye and Hill [37]	Guidance About Age-Friendly Outdoor Exercise Equipment and Associated Strategies to Maximise Usability for Older People	Journal Paper	- Outdoor exercise equipment is suitable for healthy adults and takes little account of the physical needs of older people - On-site supervision by exercise instructors should be provided to encourage older people to participate in outdoor exercise and to ensure that the equipment chosen is safe and suitable for their training.
Shedden and Kravitz [69]	Pilates Exercise: A Research-Based Review	Journal Paper	- Pilates is an effective method of body conditioning. - For older people, the springs are used to increase the training load and reverse muscle atrophy.
Roller, Kachingwe, Beling, Ickes, Cabot and Shrier [71]	Pilates Reformer Exercises for Fall Risk Reduction in Older Adults: A Randomized Controlled Trial	Journal Paper	Pilates reformer exercises significantly improve static and dynamic balance, functional mobility and active range of motion of the hip and ankle in adults over 65 years of age.
Suna and Işildak [42]	Investigation of the Effect of 8-Week Reformer Pilates Exercise on Flexibility, Heart Rate and Glucose Levels in Sedentary Women	Journal Paper	- Positive changes in physical and physiological characteristics through regular Pilates reformer training in sedentary women. - Regular exercise is important to reduce the risk of high blood pressure and diabetes, especially in older people.
Melville, Chang, Colagiuri, Marshall and Cheema [38]	Fifteen Minutes of Chair-Based Yoga Postures or Guided Meditation Performed in the Office Can Elicit a Relaxation Response	Journal Paper	- Participating in 15 min of chair-based yoga postures in the office improves physiological and psychological markers of stress. - In the long term, these practices can lead to significant health benefits, including a lower risk of cardiometabolic disease.
Kiser [72]	Decorative, Multi-Functional Exercise and Storage Bench	Patent	The unit has a storage space for storing exercise equipment such as weights, skipping ropes, water bottles, yoga mats, decorative hand weights and resistance bands.
Yu [73]	Multifunctional Exerciser	Patent	For simultaneous training of the hands, feet, lumbar spine and back of a user.
Wyatt and Wyatt [74]	Multifunctional Exercise Apparatus	Patent	Allows the user to couple one or more of the plurality of exercise media to the engagement channels, perform at least a first exercise by pulling the coupled exercise media from a contracted position to an extended position and change the orientation of the exercise media to perform at least a second exercise.
Lin [75]	Multifunctional Gym Exerciser with Adjustable Table	Patent	The user can replace the training attachments with the desired pedal unit, left leg unit and sit-up support depending on the different training requirements.
Wartes [33]	Multifunctional Pegged Furniture	Patent	Multifunctional items include an alternative chair, a step stool, a laundry basket, a two-tier table, a bench, a chest, a rocking bench, a toy box and a cradle.
Lee [30]	Multifunctional Chair	Patent	- Extended range of use. - Can be converted into a deck chair or ladder.
Tornabene, Babkes and Howell [32]	Combination Hand Truck, Stepladder, and Dolly	Patent	- Offers a versatile device for moving, lifting and supporting. - The device includes the functions of a hand lorry, a trolley and a stepladder.
Smith [31]	Ladder Top Storage Rack	Patent	Refers to fixing at the top of a ladder to keep tools and fasteners within reach with a ladder top shelf.

**Table 2:** Summary of the strengths and limitations of the main findings from previous studies.

Title and Source	Strength	Limitation
Design and Analysis of a Multifunctional Wheelchair [41] Design of a Multifunctional Wheelchair Based Fuzzy Analytical Hierarchy Process [36]	- Converts from wheelchair to bed to facilitate patient care and meet the needs of older people and people with disability. - Flexible for users who have difficulty walking, especially when they need to go to the toilet.	Might not be flexible for outdoor activities because the wheelchair is bulky and heavy.
Design and Analysis of Advanced Walker Cum Rollator [40] Design of Hybrid Rollator Cum Walker for Elderly: Review on Literature [39] Objective Measures of Rollator User Stability and Device Loading During Different Walking Scenarios [35]	- Assessment tools in fall prevention training clinics. - Several features help to reduce functional problems and increase the comfort of older people. - Rollators can be designed for older people to provide stability when walking and a sitting function to relieve stress when standing or after prolonged walking.	- May not be suitable for people who have problems with balance or weakness when standing. - The invention might take up more space compared to other simpler inventions, such as canes.
Design of Multifunctional Combined Walking Aid [68]	- Fulfils several functions such as folding and storing medicines. - Is designed for older people to ensure their safety and reduce their mobility efforts.	- May be unsuitable on wet surfaces. - May be unsafe without locking or braking mechanisms.
Objective Measures of Rollator User Stability and Device Loading During Different Walking Scenarios [35]	Rollators can serve as assessment tools in clinics for person-specific counselling and training as well as evidence-based training for fall prevention.	The product may not be suitable for people who have problems with balance or weakness when standing.
Guidance About Age-Friendly Outdoor Exercise Equipment and Associated Strategies to Maximise Usability for Older People [37]	Outdoor exercise offers older people the opportunity to participate in physical and social activities.	Outdoor exercise equipment may have restrictions set by the manufacturer regarding freedom of movement, minimum age, weight and shape or size.
Pilates Reformer Exercises for Fall Risk Reduction in Older Adults: A Randomized Controlled Trial [71]	Significantly improves static and dynamic balance, functional mobility and range of motion of the hip and ankle.	The results were not checked longitudinally to observe the sustainability of the improvements.
Investigation of the Effect of 8-Week Reformer Pilates Exercise on Flexibility, Heart Rate and Glucose Levels in Sedentary Women [42]	- Reverses muscle wasting. - Lowers the risk of high blood pressure and diabetes, especially in older people.	- Can lead to injury if the wrong posture is adopted. - Incorrect posture can reduce the effectiveness of cardio exercises.
Fifteen Minutes of Chair-Based Yoga Postures or Guided Meditation Performed in the Office Can Elicit a Relaxation Response [38]	- Fifteen minutes of yoga exercises on a chair in the office improves physiological and psychological stress markers. - Stress reduction at work has a positive effect on health.	- Not all workplaces are flexible and allow training in the office. - Incorrect postures can lead to injuries at the workplace.
Decorative, Multifunctional Exercise and Storage Bench [72] Multifunctional Exerciser [73] Multifunctional Exercise Apparatus [74] Multifunctional Gym Exerciser with Adjustment Table [75]	Facilitates the training of different parts of the body, convenient storage and different types of exercises.	Could be expensive and heavy.
Multifunctional Folding Table [34] Multifunctional Pegged Furniture [33] Multifunctional Chair [30]	Contains the functions of a deck chair, stepladder, step stool, laundry basket, two-tier table, bench, chest, rocking bench, toy box, cradle and folding table.	- Might not be very portable and flexible. - The strength of the auxiliary table function could be compromised, making it possibly less resilient than a standard table.
Combination Handtruck, Stepladder and Dolly [32]	A versatile transport, lifting and supporting device with the functions of a hand truck, dolly and a stepladder.	The design seems more suitable for industry than for domestic or other use.
Ladder Top Storage Rack [31]	A ladder to keep tools and fasteners within reach with a ladder top shelf.	Might be difficult to transport because it has a top shelf.

**Table 3:** Ideas derived from different sources.

Existing Product Ideas	Patent Ideas	Journal Ideas
Stepladder [76]	Sack truck and dolly [32]	Wheelchair stretcher [66]
Hand truck [77]	Folding chair [81]	Medical storage tank from a multifunctional combined walker [68]
Walking aid [78]	-	Wheelchair–bed [41]
Standing aid [79]	-	Pilates chair [42,71]
Chair [80]	-	-

**Table 4:** Screening of concepts using selected criteria.

Criterion	Concept 1 (Reference)	Concept 2	Concept 3	Concept 4	Concept 5
Capable of performing more than two functions	0	0	0	0	0
Capable of performing more than three functions	0	0	0	0	0
Capable of performing four functions	0	0	+	+	0
Capable of performing five functions	0	0	0	0	0
Can be easily disassembled	0	0	0	0	0
Can be folded	0	+	+	0	+
Easy to store	0	+	+	+	−
Saves time	0	+	+	+	−
Simplicity	0	−	+	−	0
Effectiveness	0	+	+	+	−
Efficiency	0	+	+	+	−
Durability	0	−	−	−	0
Total (+)	0	5	7	6	1
Total (−)	0	2	1	2	4
Net score	0	3	6	4	−3
Rank	4	3	1	2	5
Continue	no	yes	yes	yes	no

**Table 5:** Scoring of remaining concepts.

Selection Criteria	Weight (%)	Concept					
		2		3		4	
		R	Ws	R	Ws	R	Ws
Multifunctionality	20	3	15.0	4	20.0	4	20.0
Usability	20	3	15.0	3	15.0	3	15.0
Simplicity	10	2	5.0	3	7.5	3	7.5
Easy storage	10	2	5.0	4	10.0	3	7.5
Safety	20	1	5.0	2	10.0	1	5.0
Time saving	10	3	7.5	3	7.5	3	7.5
Convenience	10	3	7.5	4	10.0	4	10.0
Total	100	60.0		80.0		72.5	
Ranking		3		1		2	

## Data Availability

Not applicable.
